# Impact of COVID-19 pandemic on emergency medical system and management strategies in patients with acute coronary syndrome

**DOI:** 10.1038/s41598-023-32223-1

**Published:** 2023-03-29

**Authors:** Kohei Saiin, Sakae Takenaka, Toshiyuki Nagai, Akinori Takahashi, Yoshifumi Mizuguchi, Takao Konishi, Toshihisa Anzai, Daisuke Hotta, Mitsunori Kamigaki, Seiji Yamazaki, Tsutomu Fujita, Takehiro Yamashita, Kandoh Kawahatsu, Takashi Suzuki, Yoichi Nozaki, Taku Sakurada, Takashi Takenaka, Yasumi Igarashi, Takao Makino, The Sapporo city Acute Coronary Syndrome Network

**Affiliations:** 1 Department of Cardiovascular Medicine, Faculty of Medicine and Graduate School of Medicine, Hokkaido University, Kita 15, Nishi 7, Kita-ku, Sapporo, Hokkaido 060-8638 Japan; 2 Department of Cardiovascular Medicine, Hokkaido Cardiovascular Hospital, Sapporo, Japan; 3 Department of Cardiovascular Medicine, KKR Sapporo Medical Center, Sapporo, Japan; 4 Department of Cardiovascular Medicine, Sapporo Higashi Tokusyukai Hospital, Sapporo, Japan; 5 Department of Cardiovascular Medicine, Sapporo Cardiovascular Center, Sapporo, Japan; 6 Department of Cardiovascular Medicine, Hokkaido Ohno Memorial Hospital, Sapporo, Japan; 7 Department of Cardiovascular Medicine, Teine Keijinnkai Hospital, Sapporo, Japan; 8 Department of Cardiovascular Medicine, Kin-Ikyo Central Hospital, Sapporo, Japan; 9 Department of Cardiovascular Medicine, Hokko Memorial Hospital, Sapporo, Japan; 10 Department of Cardiovascular Surgery, Sapporo Central Hospital, Sapporo, Japan; 11 Department of Cardiovascular Medicine, NHO Hokkaido Medical Center, Sapporo, Japan; 12 Department of Cardiovascular Medicine, Sapporo-Kosei General Hospital, Sapporo, Japan; 13 Department of Cardiovascular Medicine, Sapporo City General Hospital, Sapporo, Japan

**Keywords:** Cardiology, Medical research

## Abstract

The global coronavirus disease-2019 (COVID-19) pandemic is associated with reduced rate of percutaneous coronary intervention (PCI). However, there were a few data showing how emergency medical system (EMS) and management strategies for acute coronary syndrome (ACS) changed during the pandemic. We sought to clarify changes on characteristics, treatments, and in-hospital mortality of patients with ACS transported via EMS between pre- and post-pandemic. We examined consecutive 656 patients with ACS admitted to Sapporo City ACS Network Hospitals between June 2018 and November 2021. The patients were divided into pre- and post-pandemic groups. The number of ACS hospitalizations declined significantly during the pandemic (proportional reduction 66%, coefficient −0.34, 95% CI −0.50 to −0.18, p < 0.001). The median time from an EMS call to hospital was significantly longer in post-pandemic group than in pre-pandemic group (32 [26–39] vs. 29 [25–36] min, p = 0.008). There were no significant differences in the proportion of patients with ACS receiving PCI, and in-hospital mortality between the groups. The COVID-19 pandemic had a significant impact on EMS and management in patients with ACS. Although a significant decline was observed in ACS hospitalizations, the proportion of patients with ACS receiving emergency PCI remained during the pandemic.

## Introduction

The global coronavirus disease-2019 (COVID-19) pandemic has dramatically affected the health care system. The outbreak first emerged in Wuhan, China, in December 2019^1^, and was recognised as a pandemic on 11 March 2020. In Japan, the first case of COVID-19 was reported on 15 January 2020 and the first fatality was reported on 13 February 2020. As the number of COVID-19 cases continued to increase, the Prime Minister of Japan declared a ‘State of Emergency’ for major metropolitan areas on 7 April 2020 and expanded the scope of the emergency to the entire country on 16 April 2020. The rapid increase in the number of patients with COVID-19 requiring hospitalization and intensive care had made it difficult to accept patients with other diseases that require emergency treatment.

Acute coronary syndrome (ACS), particularly ST-segment elevation myocardial infarction (STEMI), is an extremely serious disease that requires rapid transportation by the emergency medical system (EMS). Urgent coronary revascularization may be adversely affected by the COVID-19 pandemic, as the time from symptom onset to treatment and management strategies have a significant impact on patient outcomes. In the United States and Europe, the outbreak of COVID-19 has had extensive and profound effects on clinical practice, with reports of substantial drops in admissions, delayed diagnosis of health conditions, and reduction in urgent coronary revascularization rates in patients with ACS^2,3^. Notably, important regional differences exist not only in terms of the health economy, medical infrastructure, and management but also in patient characteristics. For instance, in recent years, more than 60% of urgent or emergency percutaneous coronary interventions (PCI) for patients with STEMI have been performed in Japan^4^, whereas less than 12% of all hospitals in the United States have performed PCIs for patients with STEMI, and even fewer hospitals have 24-h availability^5^. However, there is a paucity of systemic data showing how the EMS and management strategies for ACS changed during the COVID-19 pandemic.

Accordingly, this study aimed to assess changes in the number of patients transported via the EMS, the time from an EMS call to hospital arrival, treatment strategies, and in-hospital mortality in patients with ACS in an urban city of Japan before and after the COVID-19 pandemic.

## Methods

### Study design and population

The study was performed using data from the Sapporo City ACS network database collected between June 2018 and November 2021. Only the data from patients with ACS were selected and retrospectively analysed. The diagnostic and therapeutic strategies for ACS were applied by cardiologists in each hospital according to the Japanese Circulation Society guidelines for the treatment of ACS^6^. ACS included acute myocardial infarction (AMI) and unstable angina pectoris (UAP). AMI was subdivided into STEMI and non ST-segment elevation myocardial infarction (NSTEMI). Patients were classified and analysed as ACS, AMI, STEMI, and NSTEMI/UAP according to the previous report^3^.

The Sapporo City ACS network database is an ongoing multicentre registry launched in 2013 that prospectively collects information regarding emergency admissions suspected of cardiovascular emergencies to 29 acute cardiac care facilities via the EMS in Sapporo city, Japan (Supplementary Fig. S1). The Sapporo City ACS network was originally established in 2010 and jointly run by the Sapporo City Fire Department and Sapporo City Medical Association, with the goal of treating patients with emergency cardiovascular events as promptly as possible. This network covers most patients admitted via the EMS who have ACS within the area of Sapporo city, serving a population of approximately 2 million (the fifth largest population among Japanese cities). In our network system, when a patient with chest pain occurs in an area of Sapporo city, the EMS team first contacts an ACS network hospital in that district. The on-duty hospital is able to receive the patient promptly. After transport, a cardiologist, not a general physician, provides the initial response, allowing for rapid treatment of patients who need revascularization.

**Figure 1:**
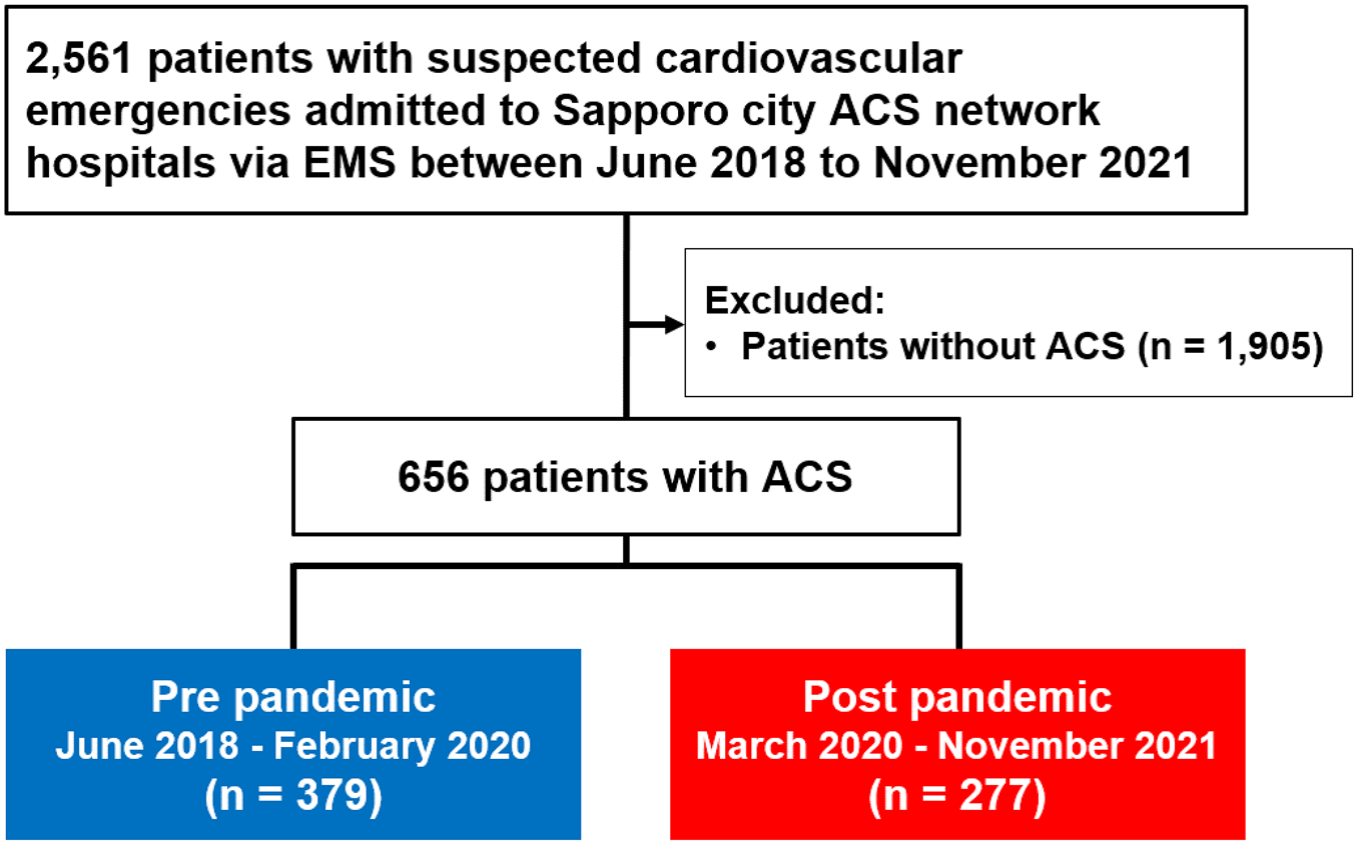
Flow diagram of the present study. ACS, acute coronary syndrome; EMS, emergency medical services.

In this study, 2561 consecutive patients registered in the Sapporo City ACS network database were identified. Of these, 1905 patients without ACS were excluded from the analysis. Ultimately, 656 patients were included in this study. In Sapporo city, the number of COVID-19 cases had increased at the end of February 2020, and ‘State of Emergency’ was declared on 28 February, ahead of the rest of Japan. Based on these circumstances, we divided our study population into pre- (before the outbreak of COVID-19, from June 2018 to February 2020) and post-pandemic (after the outbreak of COVID-19, from March 2020 to November 2021) groups (Fig. 1).

In this study, because patient information was anonymised and de-identified prior to analysis, written informed consent was not obtained from each patient. Nevertheless, we posted a summary of the protocol (with an easily understood description) at each site; the notice clearly informed the patients of their right to refuse enrolment. These procedures for informed consent and enrolment were in accordance with the detailed regulations regarding informed consent described in the guidelines, and this study, including the procedure for enrolment, has been approved by the Ethics Committee of each participating hospital^7^ (Supplementary Appendix 1 and 2) and was registered under the Japanese UMIN Clinical Trials registration (UMIN000045251). This study was conducted in accordance with the principles outlined in the Declaration of Helsinki.

### Data collection and endpoint

Individual clinical information was collected using a medical questionnaire. When an EMS team transported a Sapporo City ACS network-eligible patient, they issued a medical questionnaire that included information on the vital signs, the situation at the onset, chief complaint, and past history and passed it to a cardiologist who received the patient. After treatment, the cardiologist completed the remaining questionnaire items, including treatment details, diagnosis, and in-hospital clinical outcomes. The completed questionnaire was mailed from each hospital to the core data centre of the network at the Hokkaido University. In this study, we extracted information on demographics, medical history, clinical data, clinical course, and the use of therapeutic interventions, such as PCI, surgery, and mechanical circulatory support. The study outcomes included the following: (1) the number of patients admitted by ambulance and diagnosed with ACS, (2) time from an EMS call to hospital arrival, (3) the proportion of patients receiving coronary angiography (CAG) and emergency PCI, and (4) in-hospital mortality. The daily numbers of patients with COVID-19 were obtained from the City of Sapporo official website (City of Sapporo official website. https://www.city.sapporo.jp. Accessed on 9 May 2022)^8^.

### Statistical analyses

Continuous variables are presented as mean ± standard deviation (SD) when normally distributed and as medians and interquartile ranges (IQR) when non-normally distributed. Comparisons of differences between two groups were performed by an unpaired t-test or a Mann–Whitney U test for continuous variables and by a chi-squared test or Fisher’s exact test for dichotomous variables, when appropriate^7^. Kolmogorov–Smirnov test was used to determine whether the distribution was normal or non-normal. For variables, including the number of patients with ACS via the EMS, time from an EMS call to hospital arrival, and the number and proportion of patients with ACS receiving PCI, linear mixed effects modelling was used to determine the longitudinal changes in these variables. A two-sided *P* value < 0.05 was considered statistically significant. All data were analysed using the Stata MP64 software (version 16; StataCorp, College Station, TX, USA).

## Results

The baseline patient characteristics are presented in Table 1. The median age was 69 years [interquartile range (IQR): 58–78], 73.5% were men, and 30.1%, 70.7%, and 65.7% had diabetes mellitus, hypertension, and dyslipidemia, respectively. There were no significant differences in gender, body mass index (BMI), past history, chief complaint, Killip classification, blood pressure, or heart rate between the groups. Patients in the post-pandemic group had higher levels of serum creatinine and longer time from an EMS call to hospital arrival than those in the pre-pandemic group.

**Table 1:** Baseline patient characteristics.

Variable	All patients (N = 656)	Pre pandemic (N = 379)	Post pandemic (N = 277)	*P* value (pre vs. post pandemic)
Age (years)	69 (58—78)	68 (58—78)	70 (59—79)	0.20
Male, n (%)	477 (73.5)	271 (72.1)	206 (75.5)	0.34
Body mass index (kg/m^2^)	24.1 (22.1—27.2)	24.1 (22.1—26.9)	24.2 (22.1—27.4)	0.26
Medical history				
Diabetes, n (%)	198 (30.1)	114 (30.1)	84 (30.4)	0.92
Hypertension, n (%)	464 (70.7)	264 (69.7)	200 (72.2)	0.48
Hyperlipidemia, n (%)	430 (65.7)	247 (65.2)	183 (66.3)	0.76
Chief complaint				
Chest pain, n (%)	568 (86.6)	329 (86.8)	239 (86.3)	0.85
Dyspnoea, n (%)	35 (5.3)	18 (4.8)	17 (6.1)	0.44
Killip classification				0.110
Class Ι, n (%)	484 (82.0)	286 (85.4)	198 (77.7)	
Class II, n (%)	64 (10.8)	29 (8.7)	35 (13.7)	
Class III, n (%)	20 (3.4)	9 (2.7)	11 (4.3)	
Class IV, n (%)	22 (3.7)	11 (3.3)	11 (4.3)	
SBP (mmHg)	140 (120–160)	141 (120–160)	140 (119–159)	0.53
HR (beats/min)	73 (60–86)	72 (60–84)	76 (61–89)	0.061
Laboratory findings				
Haemoglobin (g/dL)	14.4 (12.8—15.6)	14.4 (12.6—15.6)	14.4 (12.9—15.6)	0.85
Serum creatinine (mg/dL)	0.89 (0.76—1.08)	0.87 (0.74—1.04)	0.90 (0.79—1.12)	0.021
Maximum CPK (U/L)	845 (160–2567)	757 (134–2320)	1011 (197–2819)	0.069
Maximum CK-MB (U/L)	72.2 (11.0–244.0)	60.0 (11.0–231.7)	83.2 (12.9–268.9)	0.28
ST-segment elevation, n (%)	490 (75.0)	275 (72.6)	219 (78.5)	0.085
Time from EMS call to hospital (min)	30 (25—37)	29 (25—36)	32 (26—39)	0.008

Continuous variables are presented as mean ± standard deviation if normally distributed, and median (interquartile range) if not normally distributed. Categorial variables are presented as number of patients (%).

*CK-MB* creatine kinase and its MB isoenzyme, *CPK* creatine phosphokinase, *EMS* emergency medical services, *HR* heart rate, *SBP* systolic blood pressure.

**Figure 2:**
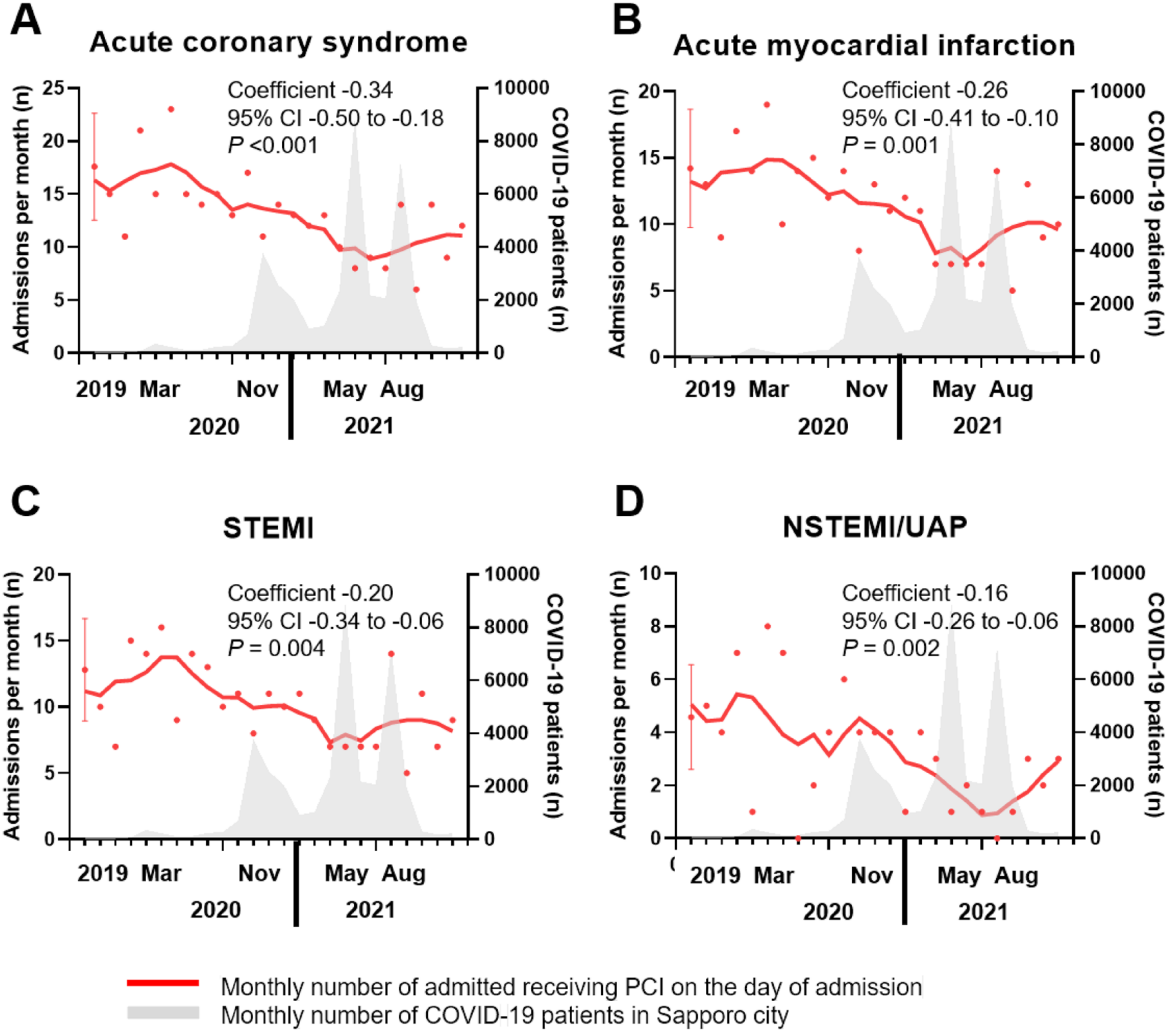
Monthly number of patients admitted via the EMS to Sapporo ACS network hospitals with acute coronary syndrome, by type. (**A**) Acute coronary syndrome, (**B**) acute myocardial infarction, (**C**) STEMI, (**D**) NSTEMI/UAP. The gray area indicates the monthly number of COVID-19 patients in Sapporo city. *COVID-19* coronavirus disease-2019, *NSTEMI* non-ST elevation myocardial infarction, *STEMI* ST elevation myocardial infarction, *UAP* unstable angina pectoris.

Reductions were recorded in entire the number of admitted patients from the average for 2019 to the end of 2021 (proportional reduction 59%, coefficient −0.86, 95% CI −1.18 to −0.54, p < 0.001) (Supplementary Fig. S2). A decline was observed in ACS admissions via the EMS between April 2021 and July 2021, with the 2019 baseline number of 17.6 admissions by ambulance per month falling to 6 per month (proportional reduction 66%, coefficient −0.34, 95% CI −0.50 to −0.18, p < 0.001) (Fig. 2A). This decline was partially reversed by October 2021. Reductions were recorded in the number of admissions by ambulance for ACS, AMI, STEMI, NSTEMI, or UAP from the average for 2019 to the end of 2021. The percentage reduction in admissions by ambulance for all AMI cases was 65%, with an average of 14.2 admissions by ambulance per month in 2019 falling to 5 per month by the end of September 2021 (coefficient −0.26, 95% CI −0.41 to −0.10, p = 0.001) (Fig. 2B). For STEMI, there were 12.8 admissions by ambulance per month in 2019 and 5 per month by the end of September 2021 (percentage reduction 61%, coefficient −0.20, 95% CI −0.34 to −0.06, p = 0.004) (Fig. 2C). The percentage reduction in admissions by ambulance for NSTEMI or UAP was 78%, with 4.6 admissions by ambulance per month in 2019 and 1 per month by the end of September 2021 (coefficient −0.16, 95% CI −0.26 to −0.06, p = 0.002) (Fig. 2D).

**Figure 3:**
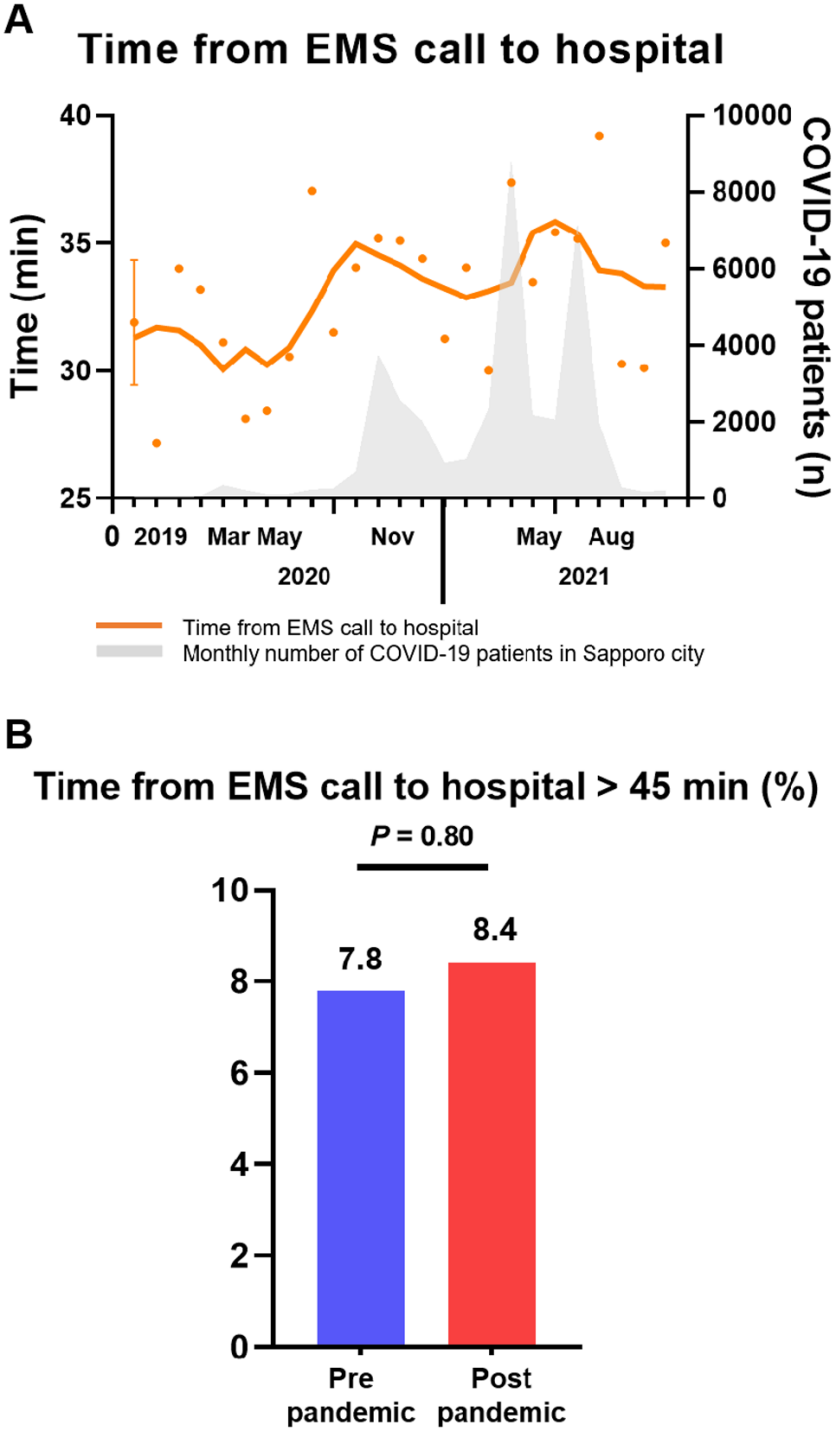
Change in the time from an EMS call to hospital arrival during the pre and post COVID-19 pandemic periods. The gray area indicates the monthly number of COVID-19 patients in Sapporo city. (**A**) Time from EMS call to hospital arrival. (**B**) Time from EMS call to hospital arrival longer than 50 min. *COVID-19* coronavirus disease-2019, *EMS* emergency medical services.

The time from an EMS call to hospital arrival was slightly longer in the post-pandemic period than in the pre-pandemic period. Our model estimated that the median time from an EMS call to hospital arrival in the pre-pandemic period was 29 (25–36) min, while it was 32 (26–39) min in the post-pandemic period (p = 0.008) (Table 1, Fig. 3A). There were no significant differences in the time from an EMS call to hospital arrival longer than 45 min between the groups (Fig. 3B).

**Figure 4:**
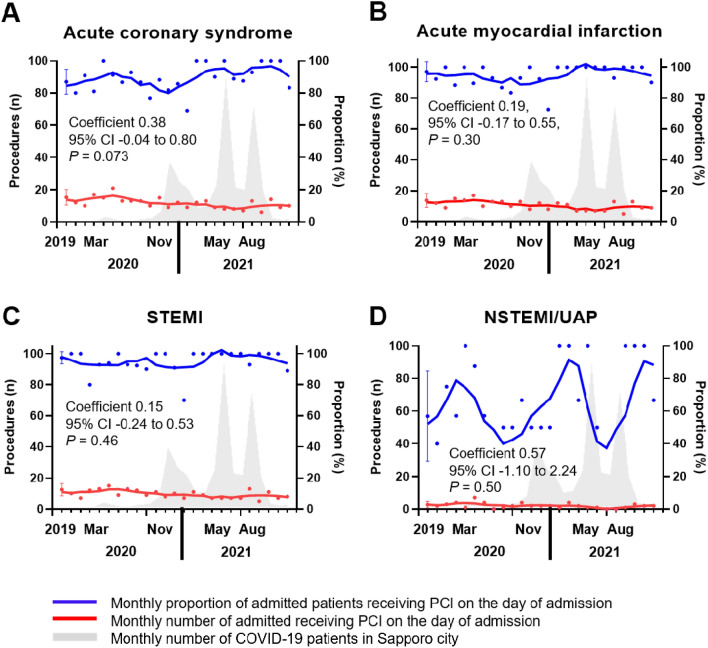
Monthly number and proportion of patients admitted via the EMS receiving PCI on the day of admission. The gray area indicates the monthly number of COVID-19 patients in Sapporo city. (**A**) Acute coronary syndrome, (**B**) acute myocardial infarction, (**C**) STEMI, (**D**) NSTEMI/UAP. *NSTEMI* non-ST elevation myocardial infarction, *PCI* percutaneous coronary intervention, *STEMI* ST elevation myocardial infarction, *UAP* unstable angina pectoris.

Procedural variables are presented in Table 2. Although the absolute number of patients in the post-pandemic period who underwent emergency CAG and PCI decreased, the proportion of patients with ACS receiving CAG and PCI on the day of admission slightly increased compared to that in the pre-pandemic period (coefficient 0.38, 95% CI −0.04 to 0.80, p = 0.073) (Fig. 4A). The reduction in admissions by ambulance for AMI and STEMI was accompanied by a slight increase in the proportion of patients admitted to the hospital and receiving PCI on the day of admission (AMI; coefficient 0.19, 95% CI −0.17 to 0.55, p = 0.30 and STEMI; coefficient 0.15, 95% CI −0.24 to 0.53, p = 0.46) (Fig. 4B,C). The proportion of patients with NSTEMI or UAP receiving PCI on the day of admission tended to decrease in response to the COVID-19 pandemic wave (coefficient 0.57, 95% CI −1.10 to 2.24, p = 0.50) (Fig. 4D). There were no significant differences between the groups in the number of patients with ACS receiving CABG (Table 2).

**Table 2:** Angiographic findings and invasive procedures.

Variable	All patients (N = 656)	Pre pandemic (N = 379)	Post pandemic (N = 277)	*P* value (pre vs. post pandemic)
Emergency CAG, n (%)	601 (91.6)	342 (90.2)	259 (93.5)	0.136
Access site				
Conventional radial approach, n (%)	356 (59.4)	192 (56.6)	164 (64.1)	0.067
Femoral approach, n (%)	237 (39.8)	146 (43.1)	91 (35.6)	0.064
Location of culprit lesion				
RCA, n (%)	239 (40.4)	132 (39.2)	107 (42.0)	0.49
LMT, n (%)	17 (2.9)	10 (3.0)	7 (2.8)	0.87
LAD, n (%)	273 (46.0)	152 (45.1)	121 (47.3)	0.60
LCX, n (%)	85 (14.4)	48 (14.2)	37 (14.5)	0.93
Treatment				
Emergency PCI, n (%)	577 (88.0)	331 (87.3)	246 (88.8)	0.57
TIMI grade 3 flow post PCI, n (%)	499 (91.4)	289 (90.6)	210 (92.5)	0.43
Door to balloon time (min)	79 (61—106)	74 (58—102)	85 (65—115)	0.002
Door to balloon time under 90 min, n (%)	320 (64.1)	194 (67.4)	126 (59.7)	0.079
CABG, n (%)	13 (2.0)	8 (2.1)	5 (1.9)	0.82
IABP, n (%)	54 (8.2)	29 (7.7)	25 (9.1)	0.52
ECMO, n (%)	9 (1.4)	5 (1.3)	4 (1.5)	0.89

Continuous variables are presented as median (interquartile range). Categorial variables are presented as number of patients (%).

*CABG* coronary artery bypass grafting, *CAG* coronary angiography, *ECMO* extra-corporeal membrane oxygenation, *IABP* intra-aortic balloon pumping, *LAD* left anterior descending artery, *LCX* left circumflex artery, *LMT* left main coronary trunk, *PCI* percutaneous coronary intervention, *RCA* right coronary artery, *TIMI* thrombolysis in myocardial infarction.

The median door-to-balloon time was 79 min (IQR 61–106). Compared to the pre-pandemic group, door-to-balloon time was longer in the post-pandemic group, but no significant difference was noted in the prevalence of door-to-balloon time within 90 min between the groups. There were no significant differences in the use of intra-aortic balloon pumping or extra-corporeal membrane oxygenation between the groups (Table 2).

Overall, in-hospital death occurred in 26 patients (3.9%), including 24 with myocardial infarction (MI), one with bleeding, and one with sepsis. There was no significant difference in the in-hospital mortality between the groups (Table 3).

**Table 3:** Clinical outcomes.

Variable	All patients (N = 656)	Pre pandemic (N = 379)	Post pandemic (N = 277)	*P* value
In-hospital death, n (%)	26 (3.9)	14 (3.7)	12 (4.3)	0.68
Cause of death				
Myocardial infarction, n (%)	24 (92.3)	12 (85.7)	12 (100)	0.173
Bleeding, n (%)	1 (3.9)	1 (7.1)	0 (0)	0.35
Sepsis, n (%)	1 (3.9)	1 (7.1)	0 (0)	0.35

Values are presented as number of patients (%).

## Discussion

This study showed the changes in the clinical characteristics, management strategies, and outcomes of patients with ACS admitted via the EMS during the COVID-19 pandemic in an urban city of Japan. The major findings were as follows: (1) a decline was seen in hospitalizations via the EMS for ACS in the post-pandemic period, (2) the time from an EMS call to hospital arrival was longer in the post-pandemic period, but remained within a few minutes difference, (3) the proportion of patients with ACS receiving CAG and PCI on the day of admission was not significantly decreased in the post-pandemic period, and (4) there were no significant differences in in-hospital mortality between the pre- and post-pandemic groups.

The COVID-19 pandemic has caused rapid changes in social, economic, and healthcare systems, and has had significant indirect impacts on the clinical course and management of patients with ACS. A study from Italy showed that the COVID-19 pandemic led to a significant increase in the proportion of myocardial infarction patients arriving at the hospital late from onset (50.0% vs 4.8%; p < 0.01) and decreased the rate of primary PCI (80.8% vs 100%; p = 0.06)^9^. In the United States and Spain, there was an estimated 40% reduction in PCI performed in patients with STEMI during the early stages of the COVID-19 pandemic^10,11^. In another survey in China, the total number of hospitalized STEMI patients nationwide declined by about 26% per week, and by about 62% in Hubei province, the epicentre of the COVID-19 outbreak. In Hubei, the median time from symptom onset to first medical contact during the COVID-19 pandemic was 6.75 (IQR 5.66–7.89) hours, compared to 5.66 (IQR 4.99–6.32) hours before the pandemic^12^. Similarly, several reports have suggested that the time for taking patients with STEMI to a hospital was significantly longer after the COVID-19 pandemic^13,14^. These delays were attributed to misled altruistic behaviour to not overburden the health care system, stay at home orders, as well as social containment mandates, and fear of COVID-19 infection^15–17^. The study showed that patients with ACS presented higher cardiac enzyme levels during the post-pandemic as compared with those in the pre-pandemic period^18^, suggesting that patients with mild symptoms would be discouraged from seeing a doctor. In fact, our patients in the post-pandemic period showed higher creatine phosphokinase levels and proportion of STEMI than those in the pre-pandemic period.

In the present study, there were no significant differences in the rates of patients with ACS receiving PCI on the day of admission and in-hospital mortality between the pre- and post- COVID-19 pandemic periods, despite the decrease in the number of patients with ACS in the post-pandemic period. These findings differ from those of previous reports from other countries. Japan has a universal health care system, and ambulance services are administered by a government-based system, with minimum fees applied to insurers. In addition, patients with STEMI may receive primary PCI in a timely manner due to a greater number of interventional cardiologists per institution in Japan^19^. Especially in the Sapporo City ACS network, a cardiovascular emergency medical network has been in operation with the help of the EMS and local medical associations in order to directly transport patients with suspected ACS to nearby PCI-capable hospitals. There are a considerable number of cardiovascular hospitals and cardiologists per population; therefore, they would be able to promptly perform emergency PCI during the COVID-19 pandemic. As a result, the rate of PCI procedures for ACS did not decrease, and in-hospital mortality did not increase in the post-pandemic period. Furthermore, we found that the COVID-19 pandemic was associated with a significantly longer time from an EMS call to hospital arrival. This may be attributed to the fact that the number of patients who were refused by the EMS increased owing to the limited number of hospitals that could accept emergency patients during the COVID-19 pandemic. Timely diagnosis and effective management of ACS are required to prevent significant morbidity and mortality, with the greatest benefit in patients presenting with ACS, especially STEMI. A previous study reported that the time from first medical contact to primary PCI is a strong predictor of adverse outcomes with every 10-min delay associated with increased mortality in patients presenting with STEMI^20^. However, our data showed that the delayed time from an EMS call to hospital arrival during the post-pandemic period was only of a few minutes, which would be acceptable in the practice for patients with ACS. In our network, the region was divided into five districts (Supplementary Fig. S1), and several hospitals in each district were on emergency duty for ACS every day. It was expected that this system would contribute to the smooth transfer of patients from the EMS to hospitals. Notably, only 4.2% of patients with ACS were transported with the time from an EMS call to hospital arrival being longer than 50 min in the post-pandemic period, which was similar to that in the pre-pandemic period, indicating that this system would show good performance.

The present study has some limitations. First, this study was limited by its retrospective design and being conducted during the pandemic, which is challenging and expected to lead to missing data. Nevertheless, the collection rate of the questionnaire before and after the pandemic was comparable. The median monthly collection rate of the questionnaire after the pandemic was 91.0% (IQR 88.6–93.8), whereas it was 91.4% (IQR 89.4–95.1) before the pandemic (p = 0.68). Second, the diagnosis and treatment of ACS were handled by each hospital, leading to significant bias. Third, we analysed only patients with ACS transported by the EMS; therefore, the studied patients did not always reflect all patients with ACS in Sapporo city. Fourth, the number of patients receiving emergency CABG was very small in this study. In Sapporo city, patients with ventricular fibrillation or cardiopulmonary arrest are generally transported to advanced emergency medical hospitals, not ACS network hospitals, which may have resulted in fewer cases with complex lesions such as a LMT lesion and multivessel disease. Finally, we could not exclude the possibility that the reduction in patients with STEMI observed in the post-pandemic period was due to the increased rate of pre-hospital death, not by the effect of the pandemic on the EMS.

## Conclusions

The COVID-19 pandemic had a significant impact on the EMS and the management of patients with ACS in an urban city in Japan. Our findings showed a substantial decline in the number of ACS admissions via the EMS, but the proportion of patients with ACS receiving emergency PCI did not decrease during the pandemic. Furthermore, the time from an EMS call to hospital arrival increased by a couple of minutes during the pandemic.

## Supplementary Information

Supplementary Information 1.

Supplementary Figures.

Supplementary Information 2.

## Data Availability

The daily numbers of patients with COVID-19 are available at https://www.city.sapporo.jp. The datasets used and/or analysed during the current study available from the corresponding author on reasonable request.
