# Supplementary material for: Impact of COVID-19 pandemic on emergency medical system and management strategies in patients with acute coronary syndrome
Source: Sci Rep. 2023 Mar 29;13:5120. doi: 10.1038/s41598-023-32223-1 (PMC10052218; doi:10.1038/s41598-023-32223-1)
Supplement: Supplementary file 1 — Supplementary Information 1. [file 41598_2023_32223_MOESM1_ESM.doc]

# Supplementary Appendix 1. Participating centres and their principal investigators

| Participating centre | Principal Investigator |
| --- | --- |
| Department of Cardiovascular Medicine, Faculty of Medicine and Graduate School of Medicine, Hokkaido University, Sapporo, Japan | Toshihisa Anzai |
| Department of Cardiovascular Medicine, Hokkaido Cardiovascular Hospital, Sapporo, Japan | Daisuke Hotta |
| Department of Cardiovascular Medicine, KKR Sapporo Medical Center, Sapporo, Japan | Mitsunori Kamigaki |
| Department of Cardiovascular Medicine, Sapporo Higashi Tokusyukai Hospital, Sapporo, Japan | Seiji Yamazaki |
| Department of Cardiovascular Medicine, Sapporo Cardiovascular Center, Sapporo, Japan | Tsutomu Fujita |
| Department of Cardiovascular Medicine, Hokkaido Ohno Memorial Hospital, Sapporo, Japan | Takehiro Yamashita |
| Department of Cardiovascular Medicine, Teine Keijinnkai Hospital, Sapporo, Japan | Kandoh Kawahatsu |
| Department of Cardiovascular Medicine, Kin-ikyo Central Hospital, Sapporo, Japan | Takashi Suzuki |
| Department of Cardiovascular Medicine, Hokko Memorial Hospital, Sapporo, Japan | Yoichi Nozaki |
| Department of Cardiovascular Medicine, Sapporo Central Hospital, Sapporo, Japan | Taku Sakurada |
| Department of Cardiovascular Medicine, The Hokkaido Medical Center, Sapporo, Japan | Takashi Takenaka |
| Department of Cardiovascular Medicine, Sapporo-Kosei General Hospital, Sapporo, Japan | Yasumi Igarashi |
| Department of Cardiovascular Medicine, Sapporo City General Hospital, Sapporo, Japan | Takao Makino |
| Department of Cardiovascular Medicine, SinSapporo Hospital of Cardiology, Sapporo, Japan | Yoichi Yamada |
| Department of Cardiovascular Medicine, Tokeidai Memorial Hospital, Sapporo, Japan | Kazushi Urasawa |
| Department of Cardiovascular Medicine, JCHO Hokkaido Hospital, Sapporo, Japan | Masashige Takahashi |
| Department of Cardiovascular Medicine, Aishin Memorial Hospital, Sapporo, Japan | Hiroshi Okamoto |
| Department of Cardiovascular Medicine, NTT East Japan Sapporo Hospital, Sapporo, Japan | Kazuyuki Noriyasu |
| Department of Cardiovascular Medicine, Hanaoka Seishu Memorial Hospital, Sapporo, Japan | Keiichi Hanaoka |
| Department of Cardiovascular Medicine, Sapporo Junkanki Hospital, Sapporo, Japan | Nobuhiro Yoshioka |
| Department of Cardiovascular Medicine, JCHO Sapporo Hokushin Hospital, Sapporo, Japan | Hiroyuki Kita |
| Department of Cardiovascular Medicine, Makomanai Memorial Sapporo Hospital, Sapporo, Japan | Eiichiro Imamura |
| Department of Cardiovascular Medicine, JR Sapporo Hospital, Sapporo, Japan | Toru Hasegawa |
| Department of Cardiovascular Medicine, Sapporo Teishinkai Hospital, Sapporo, Japan | Mamoru Hase |
| Department of Cardiovascular Medicine, Hokusei Hospital, Sapporo, Japan | Satoshi Tanazawa |
| Department of Cardiovascular Medicine, Tonan Hospital, Sapporo, Japan | Yutaka Matsui |
| Department of Cardiovascular Medicine, Tenshi Hospital, Sapporo, Japan | Mitsuhiro Nishimura |
| Department of Cardiovascular, Renal and Metabolic Medicine, Sapporo Medical University School of Medicine, Sapporo Japan | Nobuaki Kokubu |
| Department of Cardiovascular, Sapporo Orthopedics and Cardiovascular Hospital, Sapporo Japan | Takefumi Ozaki |
