# Supplementary material for: Impact of COVID-19 pandemic on emergency medical system and management strategies in patients with acute coronary syndrome
Source: Sci Rep. 2023 Mar 29;13:5120. doi: 10.1038/s41598-023-32223-1 (PMC10052218; doi:10.1038/s41598-023-32223-1)
Supplement: Supplementary file 2 — Supplementary Figures. [file 41598_2023_32223_MOESM2_ESM.pptx]

## Slide 1
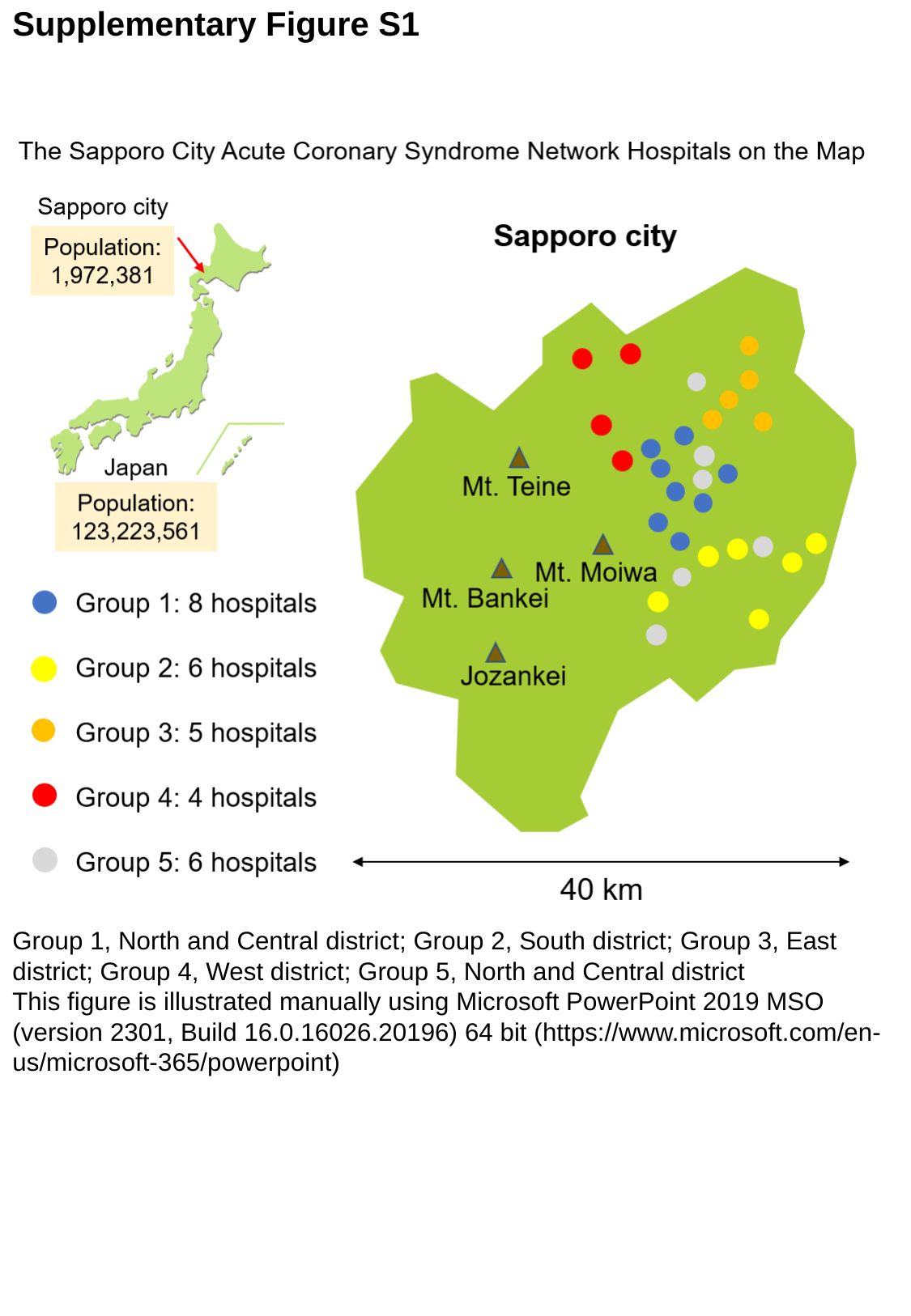

Supplementary Figure S1
Group 1, North and Central district; Group 2, South district; Group 3, East district; Group 4, West district; Group 5, North and Central district
This figure is illustrated manually using Microsoft PowerPoint 2019 MSO (version 2301, Build 16.0.16026.20196) 64 bit (https://www.microsoft.com/en-us/microsoft-365/powerpoint)

## Slide 2
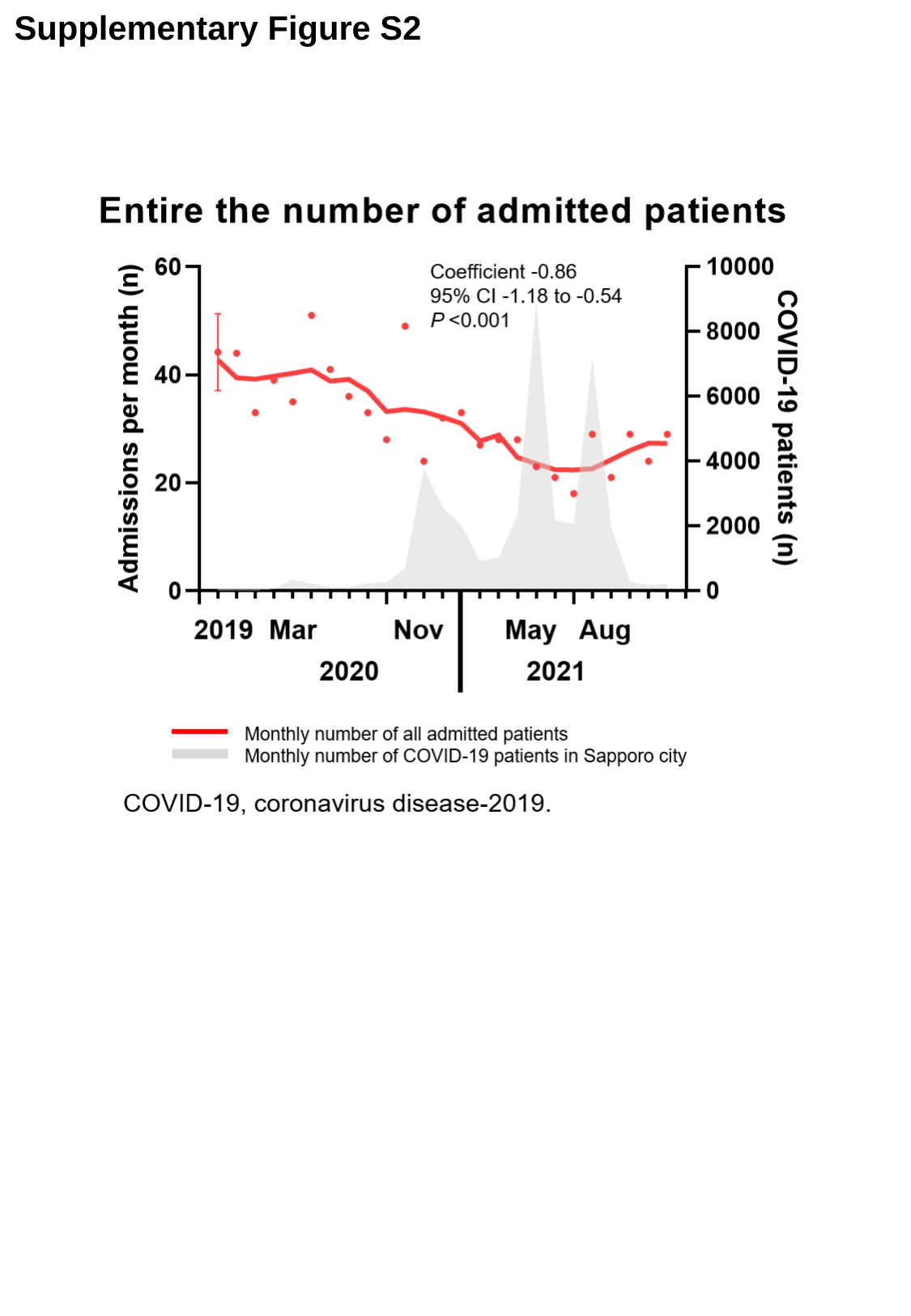

Supplementary Figure S2
