# Supplementary material for: Impact of COVID-19 pandemic on emergency medical system and management strategies in patients with acute coronary syndrome
Source: Sci Rep. 2023 Mar 29;13:5120. doi: 10.1038/s41598-023-32223-1 (PMC10052218; doi:10.1038/s41598-023-32223-1)
Supplement: Supplementary file 3 — Supplementary Information 2. [file 41598_2023_32223_MOESM3_ESM.doc]

# Supplementary Appendix 2. Participating centres and their approving committee

| Participating centre | Approving committee |
| --- | --- |
| Department of Cardiovascular Medicine, Faculty of Medicine and Graduate School of Medicine, Hokkaido University, Sapporo, Japan | Ethical Review Board for Life Science and Medical Research, Hokkaido University Hospital |
| Department of Cardiovascular Medicine, Hokkaido Cardiovascular Hospital, Sapporo, Japan | Ethics Review Board, Hokkaido Cardiovascular Hospital |
| Department of Cardiovascular Medicine, KKR Sapporo Medical Center, Sapporo, Japan | Ethics Review Board, KKR Sapporo Medical Center |
| Department of Cardiovascular Medicine, Sapporo Higashi Tokusyukai Hospital, Sapporo, Japan | Medical Ethics Review Board, Sapporo Higashi Tokusyukai Hospital |
| Department of Cardiovascular Medicine, Sapporo Cardiovascular Center, Sapporo, Japan | Crinical Ethics Review Board, Medicine, Sapporo Cardiovascular Center |
| Department of Cardiovascular Medicine, Hokkaido Ohno Memorial Hospital, Sapporo, Japan | Ethics Review Board, Hokkaido Ohno Memorial Hospital |
| Department of Cardiovascular Medicine, Teine Keijinnkai Hospital, Sapporo, Japan | Ethics Review Board, Teine Keijinnkai Hospital |
| Department of Cardiovascular Medicine, Kin-ikyo Central Hospital, Sapporo, Japan | Ethics Review Board, Kin-ikyo Central Hospital |
| Department of Cardiovascular Medicine, Hokko Memorial Hospital, Sapporo, Japan | Ethics Review Board, Hokko Memorial Hospital, Sapporo |
| Department of Cardiovascular Medicine, Sapporo Central Hospital, Sapporo, Japan | Ethics Review Board, Sapporo Central Hospital |
| Department of Cardiovascular Medicine, The Hokkaido Medical Center, Sapporo, Japan | Ethics Review Board, The Hokkaido Medical Center, Sapporo |
| Department of Cardiovascular Medicine, Sapporo-Kosei General Hospital, Sapporo, Japan | Ethics Review Board, Sapporo-Kosei General Hospital, Sapporo |
| Department of Cardiovascular Medicine, Sapporo City General Hospital, Sapporo, Japan | Ethics Review Board, Sapporo City General Hospital |
| Department of Cardiovascular Medicine, SinSapporo Hospital of Cardiology, Sapporo, Japan | Ethics Review Board, SinSapporo Hospital of Cardiology |
| Department of Cardiovascular Medicine, Tokeidai Memorial Hospital, Sapporo, Japan | Ethics Review Board, Tokeidai Memorial Hospital |
| Department of Cardiovascular Medicine, JCHO Hokkaido Hospital, Sapporo, Japan | Ethics Review Board, JCHO Hokkaido Hospital |
| Department of Cardiovascular Medicine, Aishin Memorial Hospital, Sapporo, Japan | Ethics Review Board, Aishin Memorial Hospital |
| Department of Cardiovascular Medicine, NTT East Japan Sapporo Hospital, Sapporo, Japan | Ethics Review Board, NTT East Japan Sapporo Hospital |
| Department of Cardiovascular Medicine, Hanaoka Seishu Memorial Hospital, Sapporo, Japan | Ethics Review Board, Hanaoka Seishu Memorial Hospital |
| Department of Cardiovascular Medicine, Sapporo Junkanki Hospital, Sapporo, Japan | Ethics Review Board, Sapporo Junkanki Hospital |
| Department of Cardiovascular Medicine, JCHO Sapporo Hokushin Hospital, Sapporo, Japan | Ethics Review Board, JCHO Sapporo Hokushin Hospital |
| Department of Cardiovascular Medicine, Makomanai Memorial Sapporo Hospital, Sapporo, Japan | Ethical Review Board for Life Science and Medical Research, Hokkaido University Hospital |
| Department of Cardiovascular Medicine, JR Sapporo Hospital, Sapporo, Japan | Ethics Review Board, JR Sapporo Hospital |
| Department of Cardiovascular Medicine, Sapporo Teishinkai Hospital, Sapporo, Japan | Ethics Review Board, Sapporo Teishinkai Hospital |
| Department of Cardiovascular Medicine, Hokusei Hospital, Sapporo, Japan | Ethical Review Board for Life Science and Medical Research, Hokkaido University Hospital |
| Department of Cardiovascular Medicine, Tonan Hospital, Sapporo, Japan | Ethics Review Board, Tonan Hospital |
| Department of Cardiovascular Medicine, Tenshi Hospital, Sapporo, Japan | Ethics Review Board, Tenshi Hospital |
| Department of Cardiovascular, Renal and Metabolic Medicine, Sapporo Medical University School of Medicine, Sapporo Japan | Crinical Ethics Review Board, Medicine, Sapporo Medical University School of Medicine |
| Department of Cardiovascular, Sapporo Orthopedics and Cardiovascular Hospital, Sapporo Japan | Ethics Review Board, Sapporo Orthopedics and Cardiovascular Hospital |
